# Cardioembolic stroke in Chagas disease: unraveling the underexplored connection through a systematic review

**DOI:** 10.1186/s40794-024-00227-y

**Published:** 2024-09-01

**Authors:** Jorge Vásconez-González, Camila Miño, Juan S. Izquierdo-Condoy, Camila Salazar-Santoliva, Andrés López-Cortés, Esteban Ortiz-Prado

**Affiliations:** 1 One Health Research Group, Faculty of Health Science, Universidad de Las Américas, Ecuador Calle de los Colimes y Avenida De los Granados, Quito, 170137 Ecuador; 2 Department of Public Health, London School of Hygiene and Tropical Medicine, London, WC1E 7HT UK; 3 Cancer Research Group (CRG), Faculty of Medicine, Universidad de Las Américas, Quito, Ecuador

**Keywords:** Chagas Disease, *Trypanosoma Cruzi*, Stroke risk, Cardioembolic Stroke, Cerebrovascular complications, Systematic review

## Abstract

**Background:**

Chagas disease (CD), triggered by the *Trypanosoma cruzi* parasite, is originally endemic across Latin America, affecting millions. While cardiac complications are widely recognized, the association between CD and stroke remains underexplored. This systematic review aims to elucidate the relationship between CD and stroke, highlighting the cardioembolic origins of stroke in CD patients and assessing the elevated stroke risk compared to non-infected individuals.

**Methodology:**

Adhering to the PRISMA guidelines, we conducted a comprehensive search in PubMed and Scopus databases without date restrictions, including articles in both Spanish and English. This approach enabled the identification and analysis of relevant studies to understand the interplay between CD and stroke risk.

**Results:**

Our analysis of 25 selected studies indicates that strokes in CD patients predominantly arise from cardioembolic sources. The data underscore a significant increase in stroke risk among individuals infected with *T. cruzi* compared to uninfected counterparts. Additionally, CD patients face a higher stroke and mortality risk than those with other heart failure etiologies, irrespective of disease severity.

**Conclusion:**

The review establishes CD as a critical contributor to stroke incidence, emphasizing the need for heightened awareness and diagnosis of CD in stroke patients, particularly in regions with high CD prevalence. Recognizing the increased stroke risk associated with *T. cruzi* infection is crucial for developing targeted educational and preventive strategies in endemic areas.

**Supplementary Information:**

The online version contains supplementary material available at 10.1186/s40794-024-00227-y.

## Introduction

Chagas disease (CD) and stroke represent two critical global public health challenges that collectively impose a significant morbidity and mortality burden worldwide [1–3]. CD, a neglected vector-borne disease, is caused by the protozoan parasite *Trypanosoma cruzi*, identified by Carlos Chagas in 1909 [4]. Historically, this disease predominately affected rural populations in 21 endemic countries across Latin America but has seen a geographic expansion into urban areas of non-endemic countries over the last decades due to migration and urbanization [1, 5]. The Pan American Health Organization reports that CD impacts 6–8 million people worldwide, with an estimated annual incidence of 30,000 new cases and approximately 12,000 deaths annually in the Americas [5]. 

CD’s pathogenesis involves multiple organ systems and can lead to severe, life-threatening complications necessitating ongoing treatment and monitoring [1]. The disease progresses from an acute phase, which may be asymptomatic or present with mild, nonspecific symptoms lasting 4–8 weeks, to an indeterminate phase where parasitemia becomes undetectable and may persist indefinitely if untreated. In many cases, it may develop into characteristic complications of this disease such as cardiomegaly, megaesophagus or megacolon [1, 6] Approximately 30–40% of individuals with CD will eventually exhibit chronic phase symptoms, including detectable organ damage, 10–30 years post-infection [6]. The most severe chronic complications of CD involve cardiac issues such as sudden cardiac death, heart failure, and thromboembolism, which are predominant causes of morbidity and mortality among affected individuals [1, 7]. Acute CD phase damage stems from cell-mediated immune responses targeting myocytes and the capillary endothelium, leading to muscle hyaline degeneration and coagulative necrosis [6]. Chronic Chagas cardiomyopathy, characterized by persistent inflammation-induced lesions, adversely impacts the conduction system and cardiac neural cells, culminating in significant cardiac dysfunction [1, 6]. 

Cardiac-related fatalities in CD patients are primarily due to sudden cardiac death (55–65%), progressive myocardial contraction impairment (25–30%), and stroke (10 to 15%) [6, 8]. In this context, notably, CD is implicated in up to 20% of stroke occurrences in endemic regions [8], and is recognized as a significant stroke and mortality risk factor compared to other heart failure etiologies [9]. Stroke in CD patients typically results from cardiac embolisms originating from apical ventricular aneurysms or the left atrium in cases of atrial fibrillation [3]. Additionally, aging and conventional cardiovascular risk factors such as hypertension, dyslipidemia, and smoking exacerbate prothrombotic states and endothelial dysfunction, further elevating the risk of CD-associated stroke [1, 3]. 

Given the profound health implications of the CD-stroke nexus, this systematic literature review aims to thoroughly explore stroke incidence among CD patients, addressing a gap in the literature where CD has been infrequently acknowledged as a primary stroke etiology despite its significant contribution to the global stroke burden.

## Materials and methods

### Research question

Does Chagas disease increase the risk of stroke in affected individuals?

### Study design

This systematic review synthesizes evidence from cohort studies, case-control studies, cross-sectional studies, case series, and clinical case reports. Exclusions were made for systematic reviews, literature reviews, narrative reviews, letters to the editor, editorials, and meta-analyses to ensure originality and relevance of data. The PRISMA (Preferred Reporting Items for Systematic Reviews and Meta-Analyses) guidelines were meticulously followed, providing a structured approach for this review. No review protocol was registered in PROSPERO.

### Search strategies

A bilingual (English and Spanish) literature search was conducted from January 2024 to May 2024 to encompass a broad spectrum of available evidence. Data sources included PubMed/Medline and Scopus databases, along with reference lists of pertinent articles to identify additional studies. The search was comprehensive, with no restrictions on publication date. The search strategy was based on the use of the following keywords in combination with Boolean operators: ((“Chagas disease” OR “*Trypanosoma cruzi*”) AND (“Stroke” OR “cerebrovascular disease” OR “ischemic stroke”)).

### Inclusion criteria

- Manuscripts focusing on human subjects.
- Studies mentioning Chagas disease and stroke in the title or abstract.

### Exclusion criteria

- Animal studies.
- In vivo and in vitro studies.
- Research concentrating solely on cardiac complications unrelated to stroke (e.g., heart attacks, cardiomyopathy, valvular heart disease).
- Studies addressing African trypanosomiasis and stroke.

### Bias assessment

To ensure objectivity and reduce bias, data extraction was independently conducted by JEV and JSIC at different intervals. Discrepancies were resolved through discussion until a consensus was reached, maintaining the integrity of data collection and analysis.

### Data synthesis and quality assessment

A comprehensive evaluation was undertaken on eligible manuscripts to assess the quality of included studies. For cohort and case-control studies, a quantitative synthesis was conducted utilizing the Newcastle-Ottawa Quality Assessment Scale (Tables S1 and S2, respectively). Case reports underwent appraisal using the JBI critical appraisal checklist for case reports (Table S3), while case series were evaluated with the JBI checklist for case series (Table S4), and analytical cross-sectional studies were assessed using the JBI checklist for analytical cross-sectional studies (Table S5). The application of these standardized quality assessment scales ensured a rigorous evaluation of the methodological soundness and reliability of the included studies. This process contributed to confirming that the studies encompassed in this systematic review ranged from moderate to high quality. The results and key findings were systematically organized into tables and figures to provide a comprehensive overview of the evidence synthesized.

## Results

### Literature review

**Fig. 1:**
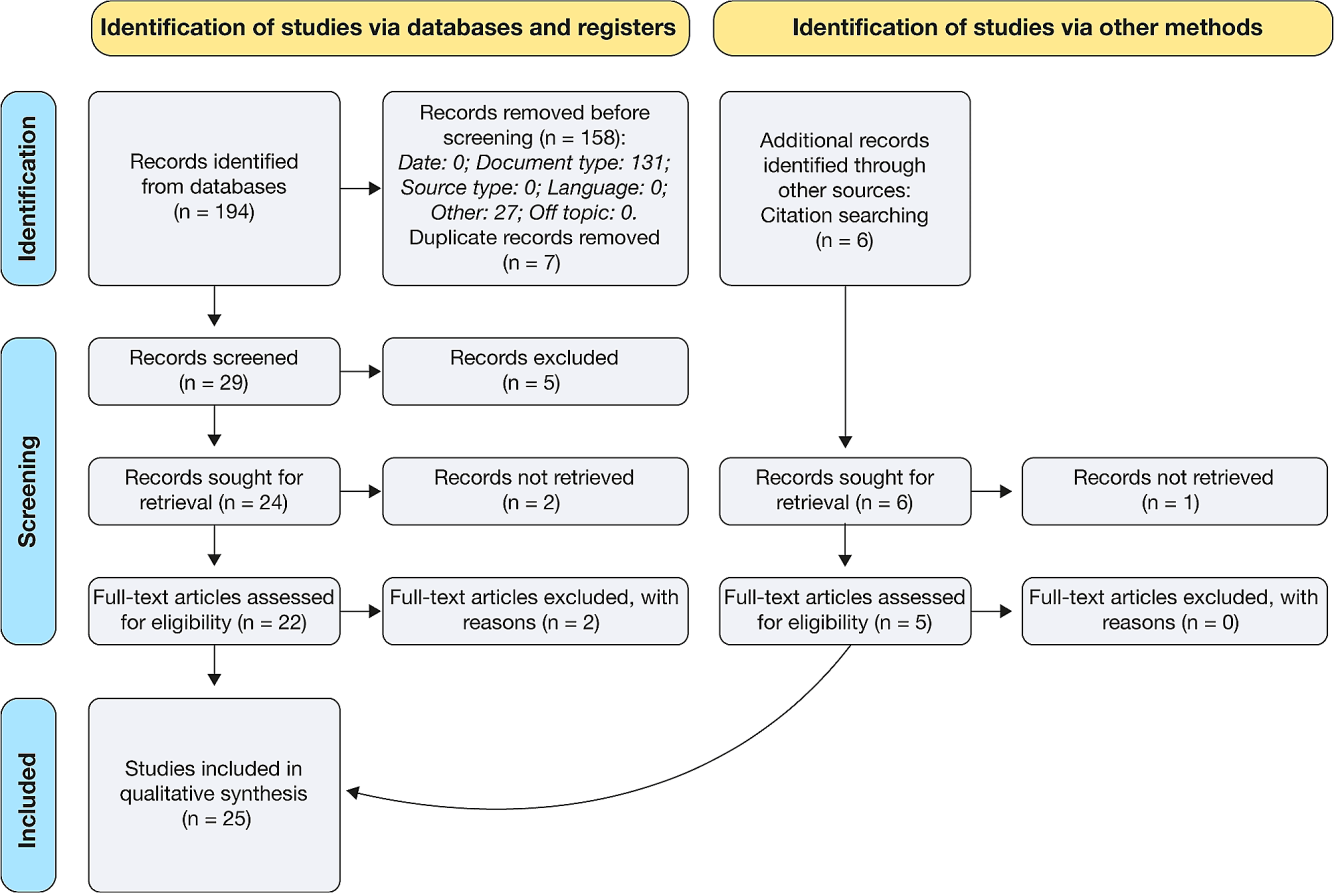
PRISMA flow diagram illustrating the literature selection process for the review

In the initial phase of the literature review process, we included 200 manuscripts. After a thorough review, 25 manuscripts were selected (Fig. 1), comprising 14 cohort studies, 5 case reports, 4 case-control studies, 1 case series, and 1 cross-sectional study (Table 1).

**Table 1:** Overview of studies on the association between Chagas disease and stroke. This table summarizes the authors, year of publication, type of study conducted, the number of participants involved, the primary objective of each study, and the key findings. The studies range from cohort and case-control designs to case reports and cross-sectional analyses, providing insights into the prevalence of encephalic infarction, the occurrence of stroke episodes, and the predictive variables for stroke in patients with Chagas Disease

Author	Year	Type of study	Participants	Objective	Result
Aras et al. [10]	2003	Cohort	5447	To determine the frequency of encephalic infarction and its contribution to lethality in patients with Chagas’ disease and heart failure.	In 524 patients with Chagas disease, brain infarction was identified in 92 (17.5%) patients. The distribution of brain infarctions according to the affected region was as follows: cerebral infarction, 89.1%; cerebellar infarction, 8.7%; and pituitary infarction, 2.17%.
Barbosa-Ferreira et al. [11]	2010	Case report	1	NA	Report of a stroke episode in a chronic chagasic patient native to the Brazilian Amazon
Bestetti [12].	2000	Cohort	79	To determine the prevalence of stroke in a hospital-derived cohort of patients with chronic Chagas’ disease.	One patient (1%) suffered a fatal stroke during the study period; he was in sinus rhythm on resting ECG and had mild mitral regurgitation, normal left ventricular function, and no intracavitary thrombus on Doppler echocardiography.
Calle-Escobar et al. [13]	2009	Case report	1	NA	Patient with ischemic stroke of probable cardioembolic origin, secondary to grade II cardiomyopathy secondary to untreated symptomatic chronic chagas disease.
Carod Artal et al. [14]	2001	Case report	2	NA	Report on 2 patients exhibiting the chronic cardiac form of Chagas disease, who experienced cardioembolic strokes.
Carod Artal et al. [15]	2003	Case series	136	To describe a group of patients with chronic or latent Chagas Disease affected by ischemic stroke and identify predictive variables for stroke in Chagas Disease patients.	Cardiomyopathy was significantly more prevalent in stroke patients with Chagas disease (45.58% vs. 24.69%; *p* = 0.00005). The diagnosis of Chagas disease was established after the presentation of a stroke in 38.23% of patients.
Carod Artal et al. [16]	2011	Case-Control	86	To identify associated vascular risk factors and stroke subtypes in ischemic stroke patients with asymptomatic T. cruzi infection and no clinical evidence of heart failure.	38.4% of chagasic stroke patients had asymptomatic T. cruzi infection. Small-vessel infarction (15.6% vs. 3.8%) and large-vessel atherosclerosis (9.4% vs. 3.8%) were significantly more frequent in asymptomatic than in symptomatic T. cruzi-infected stroke patients (*p* = 0.001).
Cerqueira-Silva et al. [9]	2022	Cohort	565	To determine the incidence of stroke and death in patients with HF, comparing Chagas and non-Chagas etiologies.	Stroke incidence was higher in Chagas compared to non-Chagas patients, with 20.2 vs. 13.9 events per 1000 patient-years, respectively. However, the death rate was similar between groups, with 41.6 for Chagas and 43.1 deaths per 1000 patient-years for non-Chagas patients.
de Matta et al. [17]	2012	Cross-sectional	chagasic (329) and non-chagasic (461) cardiomyopathies	To describe the frequency of stroke correlates in a population presenting with a cardiomyopathy, representative of a tertiary center in northeastern Brazil with a specific focus on the chagasic etiology.	In the study, there were 108 stroke cases, with a significantly higher occurrence in the Chagas group (17.3%) compared to the non-Chagas group (11.1%; *p* < 0.01). In a multivariable analysis of the entire cohort, Chagas etiology (OR = 1.79), the presence of a pacemaker (OR = 2.49), atrial fibrillation (OR = 3.03), and coronary artery disease (OR = 1.92) emerged as significant predictors of stroke.
Guedes et al. [18]	2016	Cohort	65	To evaluate the association between inflammatory markers and the risk of death and risk of stroke in patients with different clinical forms of chronic Chagas disease.	An imbalance in the inflammatory response among patients with chronic Chagas disease is linked to an elevated risk of mortality and a heightened likelihood of stroke occurrences.
Halaseh et al. [19]	2021	Case report	1	NA	An acute cerebrovascular accident, compounded by multiple prior infarctions affecting various vascular territories, was documented in a patient diagnosed with Chagas disease.
Jesus et al. [20]	2011	Cohort	144	To determine predictors of congestive heart failure in a population of patients with congestive heart failure (CHF)	Microembolic signals (MES) were identified in 9 patients (6.2%), occurring more frequently in those with Chagas disease compared to those with other causes of congestive heart failure (CHF) (12.9% vs. 1.2%, *p* = 0.005). Multivariate analysis, adjusted for age and left ventricular ejection fraction, identified Chagas disease (OR = 1.15, 95% CI: 1.05–1.26, *p* = 0.004) and a history of stroke (OR = 1.27, 95% CI: 1.05–1.26, *p* = 0.004) as predictors of MES.
Leon-Sarmiento et al. [21]	2004	Case-Control	82 cases 102 controls	To demonstrated a link between Chaga disease and symptomatic cerebrovascular disease.	In stroke cases, T. cruzi infection was significantly more prevalent (24.4%) compared to controls (1.9%), as evidenced by statistical analysis (Chi-square: 21.72; OR : 16.13; 95% CI: 3.64–71.4; *p* < 0.00001).
Lima-Costa et al. [22]	2010	Cohort	1398	To investigate the relationship between Chagas disease and long-term stroke mortality in a large community-based cohort of older adults.	Among T. cruzi-infected participants, the risk of death from stroke was twice as high as that in noninfected individuals (adjusted hazard ratio: 2.36; 95% CI: 1.25–4.44).
Melo et al. [23]	2024	Case-Control	678	To discern factors linked to stroke in Chagas disease by contrasting patients with and without a history of ischemic stroke.	Logistic regression analysis in this study identified that congestive heart failure, right bundle branch block, left anterosuperior divisional block, and atrial fibrillation are factors independently associated with an increased risk of stroke in patients with Chagas disease.
Montanero et al. [24]	2018	Cohort	279	To determine factors associated with mortality and recurrence of ischemic stroke in patients with ischemic stroke and Chagas disease.	In this study, the authors conducted a multivariate analysis to assess factors associated with mortality in stroke patients. The analysis identified several key factors: age at the time of stroke (hazard ratio [HR] 1.04), the severity of disability as measured by the initial modified Rankin Scale score (HR 20.91), the presence of bladder dysfunction (HR 2.51), diabetes mellitus (HR 3.64), and alcoholism (HR 3.37). Each of these variables was significantly associated with an increased risk of mortality, highlighting the complex interplay of health conditions that contribute to outcomes in stroke patients.
Montanero et al. [25]	2016	Cohort	86	To evaluate the etiology of stroke in patients with ischemic stroke and Chagas disease using the Trial of Org 10,172 in Acute Stroke Treatment (TOAST) and Stop Stroke Study/Causative Classification System (SSS/CCS TOAST) classification criteria.	In this study, stroke etiology was classified using two different systems, revealing significant findings. The Trial of Org 10,172 in Acute Stroke Treatment (TOAST) Classification showed that 45% of strokes had an undetermined etiology and 45% were of cardioembolic origin. In contrast, the Stop Stroke Study/Causative Classification System (SSS/CCS) TOAST results differed slightly, indicating that 34% of strokes were undetermined, while 50% were identified as cardioembolic (*p* < 0.01). This discrepancy underscores the complexity of diagnosing stroke origins and the importance of employing multiple classification systems for a comprehensive understanding.
Montanero et al. [26]	2019	Cohort	279	To describe the epidemiological and geographical distribution of IS in Chagas disease in patients attending multicenter, open access, quaternary rehabilitation hospital network spread across four of the five regions of Brazil.	In this investigation, a stark contrast was observed in the distribution of stroke cases across different socio-economic settings. Cities characterized by lower income levels and diminished access to healthcare facilities reported the highest incidence of stroke cases. Furthermore, the study highlighted a variable distribution of vascular risk factors and outcomes post-stroke among the units, suggesting that socio-economic factors significantly influence stroke prevalence and patient recovery trajectories.
Montanero et al. [27]	2021	Cohort	499	To describe a multicenter cohort of patients with concomitant CD and IS admitted in tertiary centers and to create a predictive model for cardioembolic embolism in CD and IS.	In this study, the development of a predictive model for the etiological classification of stroke in Chagas disease revealed a close correlation with the number of abnormalities detected on echocardiography and electrocardiography. Additionally, the model demonstrated a significant association with vascular risk factors, underscoring the importance of these indicators in predicting the likelihood of stroke in patients with Chagas disease.
Montanero et al. [28]	2024	Cohort	499	To describe the largest cohort of patients with Chagas disease and ischemic stroke and determining variables associated with stroke recurrence and cardioembolic cause.	In the investigation, it was discovered that there was a 29.7% recurrence rate of stroke, notably higher within the cardioembolic subgroup. Furthermore, 56% of the patients experienced embolic strokes of undetermined origin. The study also identified specific electrocardiogram (EKG) abnormalities that were linked to an increased risk of a stroke having a cardioembolic etiology.
Nunes et al. [29]	2009	Cohort	213	To describe the incidence and to evaluate the effect of LV ejection fraction on the risk for ICE in patients with Chagas cardiomyopathy.	The study reported an overall incidence of ischemic cerebrovascular events (ICE) at 2.67 events per 100 patient-years. Analysis revealed independent risk factors for ICE to include left ventricular (LV) ejection fraction (HR = 0.95, 95% CI 0.91 to 0.99, *p* = 0.009) and left atrial volume corrected for body surface area (HR = 1.04, 95% CI 1.01 to 1.07, *p* = 0.007).
Nunes et al. [30]	2015	Cohort	330	To assess the prevalence of ischemic cerebrovascular events (ICE) among patients with Chagas heart disease and to identify the risk factors associated with cardioembolism in this population.	In this study, multivariate analysis identified apical aneurysm (aOR 2.19, 95% CI 1.11 to 4.34; *p* = 0.024) and LV thrombus (aOR 2.43, 95% CI 1.09 to 5.42; *p* = 0.030) as significant determinants of ischemic cerebrovascular events (ICE), even after adjusting for anticoagulation therapy.
Nussenzveig et al. [31]	1953	Case report	8	NA	In this study, eight cases of Chagas’ heart disease were documented, all confirmed by a positive Machado-Guerrero reaction. These patients experienced cerebrovascular accidents during their disease.
Oliveira-Filho et al. [32]	2005	Cohort-	305	To identify the predictors of stroke in a region where Chagas Disease is endemic.	In this investigation, the occurrence of stroke was significantly higher in patients with CD, at 15.0%, compared to those with other cardiac conditions, which had a stroke rate of 6.3% (*p* = 0.015).
Paixão et al. [33]	2009	Case-Control	101 cases 100 controls	To investigate Chagas Disease, defined by positive serology, as an independent risk factor for stroke.	In this study, the authors demonstrated that after conducting a multivariable analysis using a backward elimination procedure, a history of previous stroke/transient ischemic attack (OR = 6.98; 95% CI, 2.99 to 16.29), atrial fibrillation (OR = 4.52; 95% CI, 1.45 to 14.04), and positive serology for CD (OR = 7.17; 95% CI, 1.50 to 34.19) remained as independent risk factors associated with stroke.

In the realm of medical research, stroke has emerged as a critical concern in patients with CD, manifesting in approximately 10–20% of individuals diagnosed with this condition [34]. A notable study conducted in Colombia highlighted that ischemic stroke occurs with greater frequency in individuals infected with *T. cruzi* (24.4%) compared to those uninfected (1.9%) [21]. Furthermore, CD is significantly associated with an increased risk of both stroke (*p* = 0.048) and mortality (*p* = 0.037) relative to other heart failure etiologies, irrespective of severity [9]. Key risk factors for ischemic stroke in CD patients include left ventricular (LV) ejection fraction and left atrial volume corrected for body surface area, even after adjusting for anticoagulation therapy [23, 29]. 

Additionally, in an extensive autopsy study involving 5445 cases, of which 524 had CD, cerebral infarction was the predominant form of brain infarction observed (89.1%), followed by cerebellar (8.7%) and pituitary infarction (2.17%) [10]. Barbosa et al. and Calle et al. reported on male patients over 30 with CD and cardioembolic stroke, uncovering various types of blocks on electrocardiograms and confirming chronic CD in all cases [11, 13]. An epidemiological study in Brazil revealed that 75% of ischemic stroke patients did not exhibit acute forms of Chagas infection [26]. Carod et al. determined the primary etiology of strokes as cardioembolism (52.2%), with other cases being undetermined, atherothrombotic, or resulting from small vessel disease [15]. For their part, Montanaro et al. found similar patterns, classifying 45% of ischemic strokes in their study as cardioembolic [25]. Historical data from 1953 first documented cardioembolic origin strokes in CD patients, emphasizing the significance of apical aneurysm and intracavitary thrombus as determinants for embolic stroke development [30]. In patients experiencing congestive heart failure, the presence of CD significantly increases the risk for the development of silent cerebral microembolisms (*p* = 0.004), a risk that persists irrespective of the heart disease’s severity. Additionally, abnormalities detected in the electrocardiogram are linked to an elevated risk of cardioembolic strokes [20, 28]. 

In a predictive model, a close association was identified between the number of anomalies detected in echocardiography and electrocardiography (> 3) and vascular risk factors (diabetes and hypertension), indicating an increased likelihood of experiencing a cardioembolic-origin stroke [27]. 

In cases of non-embolic etiology strokes, inflammation plays a pivotal role; the inflammatory response is intricately linked to immune modulation in the progression of ischemic stroke [35]. Data indicate that an inflammatory imbalance in patients with chronic Chagas disease (CD) is associated with a higher risk of death and stroke. Notably, reduced expression of transcription factors (GATA-3, FoxP3), cytokine IL-10, and elevated mRNA expression of IFN-γ, TNF-α, and inducible nitric oxide synthase (iNOS) have been observed to increase the risk of death from stroke in these patients patients [18]. This trait is particularly evident in patients exhibiting the cardiac form of the disease compared to those with the indeterminate form. Patients with the cardio-digestive form also showed higher expression of TNF-α compared to those with the indeterminate form of the disease [18]. A case-control study conducted in Brazil revealed that 38.4% of Chagas stroke patients presented asymptomatic *T.cruzi* infection. Moreover, small vessel infarction (15.6%) and large vessel atherosclerosis (9.45%) were more prevalent in this group compared to symptomatic stroke patients with CD (3.8%) [16]. 

In a cohort of 9749 individuals aged over 60 years monitored for a decade, 37.5% were found to be infected by *T. cruzi*, and among these patients, the mortality rate from stroke was twice as high as that of the uninfected. Furthermore, elevated levels of type B natriuretic peptide and electrocardiographic atrial fibrillation were shown to increase the risk of stroke mortality by 11.49 times in infected individuals [22]. Comparing patients with Chagas cardiomyopathy to those without revealed that stroke occurrences were more frequent in patients with CD (17.3% vs. 11.1%; *p* < 0.01). It was also noted that strokes occurred with higher frequency without any vascular risk factors in the Chagas cohort compared to the non-Chagas cohort [17]. 

When comparing CD in patients with cerebrovascular accidents to those with acute coronary syndrome, positive CD serology (*p* = 0.002) was more common among stroke patients. Furthermore, multivariate analysis indicated that positive serology for CD (OR = 7.17; 95% CI, 1.50 to 34.19) was independently associated with stroke [33]. Stroke was also found to be more prevalent in CD (15.0%) compared to other heart diseases (6.3%; *p* = 0.015), with CD being a predictor of stroke in multivariate analysis (OR = 1.09; 95% CI, 1.02 to 1.17) [32]. Montanaro et al. analyzed stroke recurrence in patients with CD, identifying associated factors such as age at the time of stroke (OR = 0.96), cognitive deficit (OR = 0.44), initial modified Rankin Scale (OR = 1.84), cardioembolic etiology (OR = 2.47), and female sex (OR = 2.73) [24].

## Discussion

The evidence compiled in this review underscores a pronounced vulnerability among individuals harboring the *T. cruzi* parasite, responsible for CD, towards experiencing cerebrovascular incidents. Particularly, those exhibiting the chronic manifestation of the disease manifest an escalated risk for strokes, independent of their cardiac functionality [36]. Notably, among patients encountering a cerebrovascular event, a substantial fraction, up to 30%, tested positive for CD, with this proportion surging to 62.5% in individuals afflicted by both CD and a cerebrovascular incident [37]. 

This correlation suggests a heightened propensity for stroke development in *T. cruzi*-infected individuals, particularly within those presenting the chronic form of CD. Predominantly, strokes in these patients are cardioembolic. Literature supports the linkage between CD and embolic-origin strokes, attributing such occurrences to endothelial damage augmentation, diminished blood circulation, and a dysregulation in coagulation factors conducive to thrombus formation [13, 15–30, 14, 19, 38]. 

Corad & Gascon delineated key risk factors for cardioembolic stroke within CD patients, categorizing them into three groups: (1) Cardiomyopathy, including left atrium dilation, progressive heart failure (systolic or diastolic), segmental lesions (left ventricular posterior wall lesion, apical aneurysm); (2) Arrhythmias, specifically right bundle branch block with left anterior hemiblock, advanced atrioventricular block, atrial fibrillation, and sustained ventricular tachycardia; and (3) Mural thrombus. Apical aneurysms (37.23%), left ventricular dilation (23.4%), and mural thrombus (11.7%) are identified as prevalent manifestations [37, 39]. 

Reports by Barbosa et al. and Calle et al. noted electrocardiogram (EKG) alterations in their cases [11, 13] with approximately 70% of Chagas stroke patients displaying EKG anomalies, including right bundle branch block, left fascicular block, and atrial fibrillation. Such findings, particularly in regions endemic to Chagas, could serve as early warning indicators, enabling healthcare professionals to anticipate and strategize potential stroke interventions and prophylaxis for Chagas patients [40]. 

Beyond cardioembolic sources, literature also acknowledges non-cardioembolic mechanisms, where chronic inflammation plays a critical role. The infection initiates macrophage activation and iNOS production, infringing upon the vascular endothelium. This cascade, marked by an excessive nitric oxide release due to heightened iNOS activity, potentially suppresses endothelial nitric oxide synthase activity, culminating in vasoconstriction, cerebral microvascular spasms, and ultimately, ischemic strokes. Other implicated causes include large vessel atherothrombosis (8.5%) and small vessel disease (9.6%) [40–42]. 

**Fig. 2:**
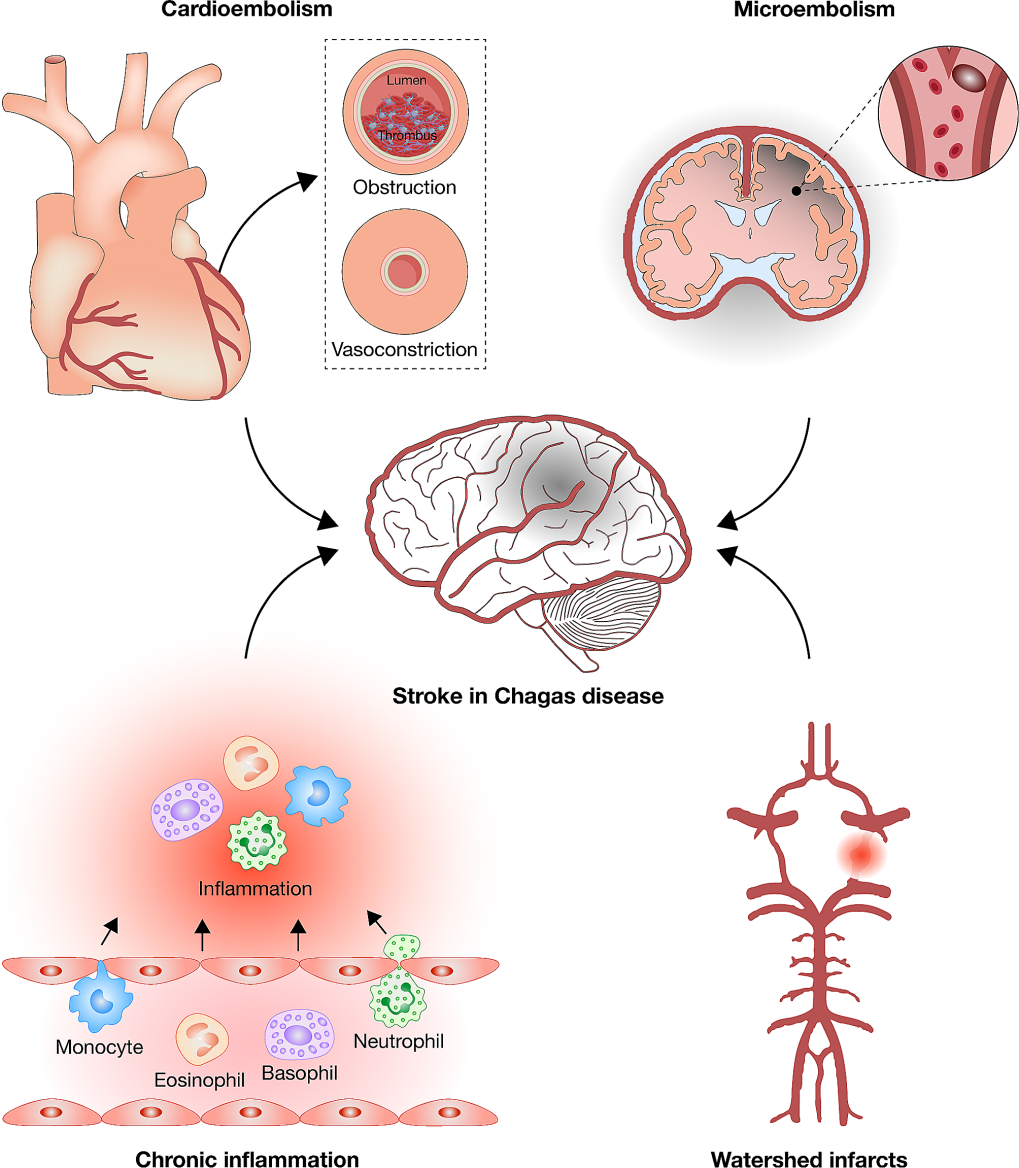
Mechanisms of Ischemic Stroke Development in Patients with Chagas Disease (CD). This figure delineates the four primary mechanisms implicated in the onset of ischemic stroke in individuals diagnosed with CD: cardioembolisms, microembolisms, chronic inflammation, and watershed infarcts. Each mechanism is a potential pathway through which CD contributes to the increased risk of ischemic stroke, highlighting the complexity of interactions between the disease and cerebrovascular events [34]

In patients with CD, four mechanisms implicated in the development of ischemic stroke are identified (Fig. 2): cardioembolisms, microembolisms, chronic inflammation, and watershed infarcts [34]. 

**Fig. 3:**
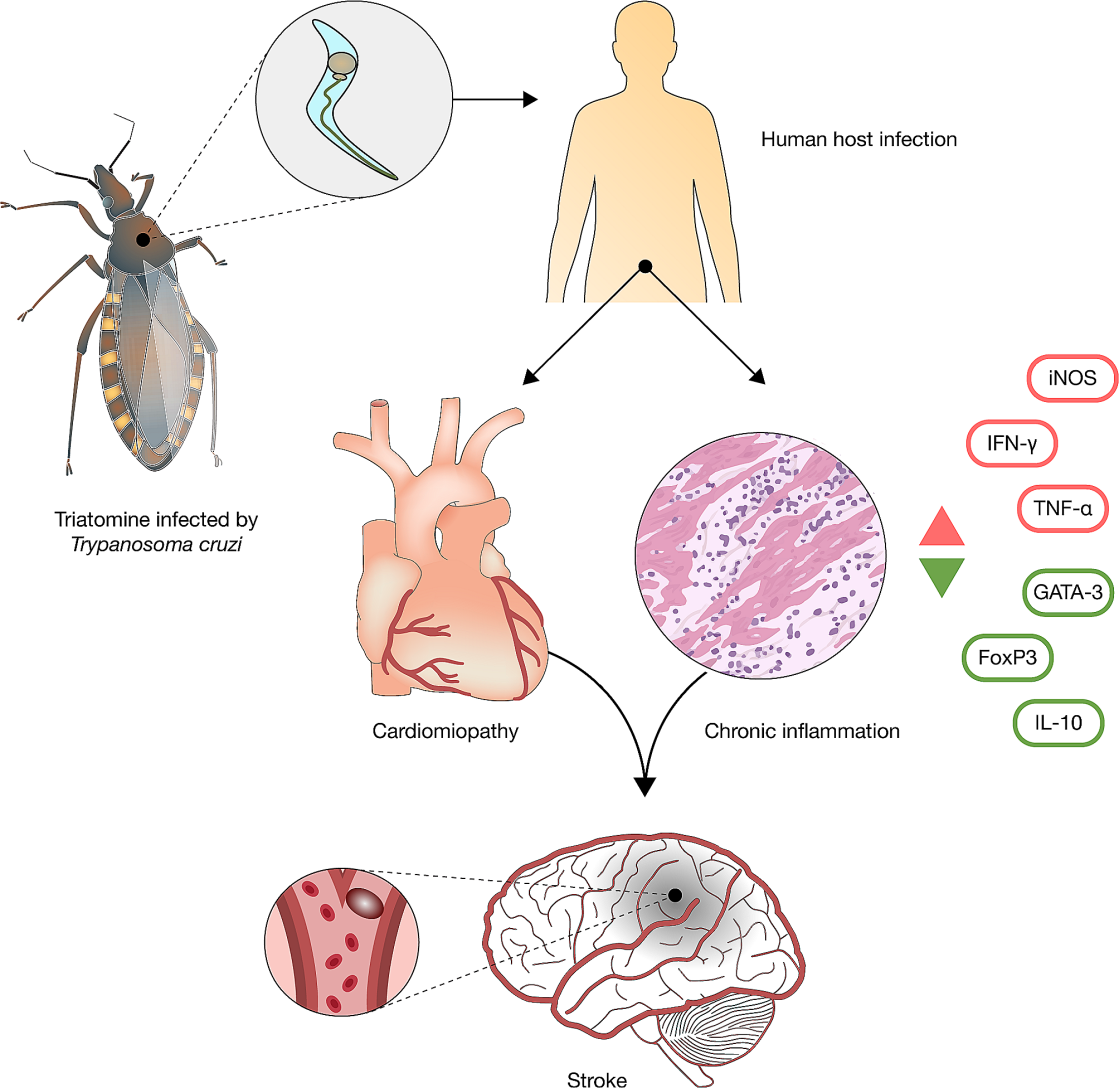
Pathways of Increased Ischemic Stroke Risk in Chagas Disease (CD) Patients due to T. cruzi infection. The figure delineates two main mechanisms: structural cardiac alterations by T. cruzi leading to cardioembolic events, and a T. cruzi-triggered immune response causing chronic inflammation that promotes atherosclerosis. Guedes et al. identified an immune imbalance in CD patients—lower regulatory cytokines (GATA-3, FoxP3, IL-10) and higher pro-inflammatory markers (IFN-γ, TNF-α, iNOS)—which correlates with an increased stroke risk and mortality. This immune dysregulation accelerates atherosclerosis, linking CD with ischemic stroke, further evidenced by studies showing a relationship between altered cytokine levels and atherosclerosis progression in CD [34]

Oliveira-Filho, recognizing that existing theories fail to account for the specific nature of brain involvement observed in CD patients, argues for the necessity of a new theoretical framework [34]. He suggests that brain involvement in CD may principally stem from two mechanisms, as depicted in Fig. 3.

The first mechanism involves structural cardiac damage induced by *T. cruzi*, while the second, a concurrent mechanism, pertains to the immune response to *T. cruzi*, characterized by chronic inflammation that can accelerate atherosclerosis, thereby increasing the risk of ischemic stroke. Guedes et al. have noted an imbalance in the immune response among CD patients, marked by a predisposition towards stroke and higher mortality rates. This imbalance is characterized by decreased levels of GATA-3, FoxP3, and IL-10, coupled with increased levels of IFN-γ, TNF-α, and iNOS [18]. Further studies have corroborated these findings, indicating that elevated levels of TNF-γ and IFN-γ, alongside reduced levels of IL-10 in individuals with Chagas disease, may hasten the progression of atherosclerosis and subsequently lead to ischemic stroke [43–45]. 

This systematic review is one of first of its kind on this subject. During its execution, we did not find any other systematic reviews published on this topic in the databases used. This review aims to elucidate the complex relationship between stroke and Chagas disease (CD), with the goal of informing and guiding clinical practice and research. Despite the insights gained, numerous aspects of this relationship remain unclear, emphasizing the necessity for further, well-controlled studies to unravel the multifaceted interactions at play. The findings underscore an urgent need for a deeper investigation into the mechanisms underlying stroke in CD, to refine preventative strategies and therapeutic interventions for affected populations.

### Limitations

This systematic review rigorously adheres to PRISMA guidelines but encounters limitations including methodological heterogeneity among included studies, potentially biasing outcomes and affecting generalizability. Language restrictions might have excluded pertinent studies, limiting the evidence base. Additionally, the exclusion of gray literature and studies focusing solely on cardiac complications unrelated to stroke could contribute to publication bias and overlook interconnected cardiovascular insights. The absence of analyses on ethnic, gender and environmental factors further restricts the review’s applicability across diverse populations. Outcome assessment heterogeneity complicates definitive conclusions regarding CD and stroke risk. These factors necessitate cautious interpretation of the findings and underscore the need for further research to address these gaps, standardize outcomes, and enhance our understanding of CD’s impact on stroke, informing clinical and public health practices.

## Conclusions

The review positions CD as a pivotal factor in the increased incidence of strokes, underscoring the imperative for enhanced awareness and prompt diagnosis of CD among stroke patients, especially in regions where CD is highly prevalent. It is essential to acknowledge the elevated risk of stroke linked to *T. cruzi* infection to foster the development of focused educational and preventive measures within endemic zones. This approach aims to mitigate the impact of CD on stroke prevalence by facilitating early detection, implementing secondary prevention tactics, and ensuring healthcare professionals are adept at identifying and managing the disease’s neurological complications. The strategic emphasis on education, coupled with robust vector control and screening programs, will be crucial in reducing the global health burden of CD and its consequential risk of stroke, particularly in remote and underserved areas. This concerted effort requires a comprehensive understanding of CD’s role in stroke etiology, advocating for an integration of targeted interventions to improve patient outcomes and decrease the incidence of stroke associated with CD.

### Recommendations

Future research should focus on investigating variables such as the duration since *T. cruzi* infection or its diagnosis and the frequency and severity of cerebrovascular events. Additionally, examining patient ethnicity, gender and residential altitude could unveil key determinants of stroke susceptibility and outcomes in Chagas disease, informing targeted interventions and preventive measures.

### Electronic supplementary material

Below is the link to the electronic supplementary material.

Supplementary Material 1

## Data Availability

No datasets were generated or analysed during the current study.

## Abbreviations

CD: Chagas disease
PRISMA: Preferred Reporting Items for Systematic Reviews and Meta-Analyses
