# Supplementary material for: Cardioembolic stroke in Chagas disease: unraveling the underexplored connection through a systematic review
Source: Trop Dis Travel Med Vaccines. 2024 Sep 1;10:16. doi: 10.1186/s40794-024-00227-y (PMC11366139; doi:10.1186/s40794-024-00227-y)
Supplement: Supplementary file 1 — Supplementary Material 1 [file 40794_2024_227_MOESM1_ESM.docx]

**Supplementary material**

**Table S1:** Newcastle - Ottawa quality assessment scale.

| **Study** | **1** | **2** | **3** | **4** | **5** | **6** | **7** | **8** | **Score** |
| --- | --- | --- | --- | --- | --- | --- | --- | --- | --- |
| Aras et al ^14^ | * |  | * | * | ** | * | * | * | 8 |
| Bestetti ^32^ | * |  | * | * | * | * | * | * | 7 |
| Cerqueira-Silva et al. ^9^ | * |  | * | * | ** | * | * | * | 8 |
| Guedes et al. ^25^ | * |  | * | * | * | * | * | * | 7 |
| Jesus et al.^21^ | * |  | * | * | ** | * | * | * | 8 |
| Lima-Costa et al. ^27^ | * |  | * | * | * | * | * | * | 8 |
| Montanero et al. ^31^ | * |  | * | * | ** | * | * | * | 8 |
| Montanero et al.^19^ | * | * | * | * | ** | * | * | * | 9 |
| Montanero et al.^17^ | * | * | * | * | ** | * | * | * | 9 |
| Montanero et al. ^23^ | * |  | * | * | * | * | * | * | 7 |
| Montanero et al. ^22^ | * |  | * | * | * | * | * | * | 7 |
| Nunes et al. ^12^ | * |  | * | * | ** | * | * | * | 8 |
| Nunes et al. ^20^ | * | * | * | * | * | * | * | * | 8 |
| Oliveira-Filho et al ^30^ | * | * | * | * | ** | * | * | * | 9 |

**Note:** A study can receive a maximum of one star for each item numbered within the Selection and Result categories. A maximum of two stars can be awarded for comparability.

**Selection**

1. Representativeness of the exposed court.

2. Selection of the unexposed court.

3. Exposure determination.

4. Demonstration that the current outcome of interest was not present at baseline.

**Comparability**

5. Cohort comparability based on design or analysis.

**Results**

6. Evaluation of the result.

7. Was the follow-up long enough for the results to occur?

8. Adequacy of cohort follow-up.

**Interpretation**

Good quality: 3 or 4 stars in the selection domain and 1 or 2 stars in the comparability domain and 2 or 3 stars in the outcome/exposure domain.

Acceptable quality: 2 stars in the selection domain and 1 or 2 stars in the comparability domain and 2 or 3 stars in the outcome/exposure domain.

Poor quality: 0 or 1 star in the selection domain or 0 stars in the comparability

domain or 0 or 1 stars in the outcome/ exposure domain

**Table S2:** Newcastle - Ottawa quality assessment scale.

| **Study** | **1** | **2** | **3** | **4** | **5** | **6** | **7** | **8** | **9** | **10** | **Score** |
| --- | --- | --- | --- | --- | --- | --- | --- | --- | --- | --- | --- |
| Carod Artal et al.^26^ | * |  | * | * | * | * | * | * |  | * | 8 |
| Leon-Sarmiento et al. ^11^ | * |  | * | * | * | * | * | * |  | * | 8 |
| Melo et al.^13^ | * |  | * | * | * | * | * | * | * | * | 9 |
| Paixão et al. ^29^ | * |  | * | * | * | * | * | * | * | * | 9 |

**Is the Case Definition Adequate?**

1. Requires some independent validation (e.g. >1 person/record/time/process to

extract information, or reference to primary record source such as x-rays or

medical/hospital records)

2. Record linkage (e.g. ICD codes in database) or self-report with no reference to

primary record

3. No description

**Representativeness of the Cases**

4. All eligible cases with outcome of interest over a defined period of time, all cases

in a defined catchment area, all cases in a defined hospital or clinic, group of

hospitals, health maintenance organization, or an appropriate sample of those

cases (e.g. random sample)

5. Not satisfying requirements in part (a), or not stated.

**Selection of Controls**

6. Community controls (i.e. same community as cases and would be cases if had

outcome)

7. Hospital controls, within same community as cases (i.e. not another city) but

derived from a hospitalized population

8. No description

**4 Definition of Controls**

9. If cases are first occurrence of outcome, then it must explicitly state that controls

have no history of this outcome. If cases have new (not necessarily first)

occurrence of outcome, then controls with previous occurrences of outcome of

interest should not be excluded.

10. No mention of history of outcome

**Table S3:** JBI Critical Appraisal Checklist for Case Reports

| **Study** | **1** | **2** | **3** | **4** | **5** | **6** | **7** | **8** | **Score** |
| --- | --- | --- | --- | --- | --- | --- | --- | --- | --- |
| Barbosa-Ferreira et al. ^15^ | Yes | Yes | Yes | Yes | Yes | Yes | Yes | Yes | 8 |
| Calle-Escobar et al.^16^ | No | Yes | Yes | Yes | Yes | Yes | Yes | Yes | 7 |
| Carod Artal et al. ^33^ | Yes | Yes | Yes | Yes | Yes | Yes | Yes | Yes | 8 |
| Halaseh et al ^34^ | Yes | Yes | Yes | Yes | Yes | Yes | Yes | Yes | 8 |
| Nussenzveig et al. ^35^ | No | Yes | Yes | Yes | Yes | Yes | Yes | Yes | 7 |

1. Were patient demographics clearly described?

2. Was the patient's history clearly described and presented as a timeline?

3. Was the patient's current clinical condition clearly described at the time of presentation?

4. Were diagnostic tests or evaluation methods and results clearly described?

5. Were interventions or treatment procedures clearly described?

6. Was the post-intervention clinical condition clearly described?

7. Were adverse (harm) or unforeseen events identified and described?

8. Does the case report provide lessons to take away?

**Table S4:** JBI Critical Appraisal Checklist for Case Series

| **Study** | **1** | **2** | **3** | **4** | **5** | **6** | **7** | **8** | **9** | **10** | **Score** |
| --- | --- | --- | --- | --- | --- | --- | --- | --- | --- | --- | --- |
| Carod Artal et al.^18^ | Yes | Yes | Yes | Yes | Yes | Yes | Yes | Yes | Yes | Yes | 10 |

1. Were there clear criteria for inclusion in the case series?

2. Was the condition measured in a standard and reliable way for all participants included in the case series?

3. Were valid methods of condition identification used for all participants included in the case series?

4. Did the case series include consecutive participants?

5. Did the case series have complete inclusion of participants?

6. Were there clear reports on the demographics of the study participants?

7. Was there a clear reporting of the clinical information of the participants?

8. Were outcomes or case follow-up results clearly reported?

9. Was there clear reporting of demographic information from the presenting sites/clinics?

10. Was the statistical analysis appropriate?

**Table S5:** JBI critical appraisal checklist for analytical cross-sectional studies

| **Study** | **1** | **2** | **3** | **4** | **5** | **6** | **7** | **8** | **Score** |
| --- | --- | --- | --- | --- | --- | --- | --- | --- | --- |
| de Matta et al. ^28^ | Yes | Yes | Yes | Yes | Yes | No | Yes | Yes | 7 |

1. Were the criteria for inclusion in the sample clearly defined?

2. Were the study subjects and setting described in detail?

3. Was the exposure measured in a valid and reliable way?

4. Were standard and objective criteria used to measure the condition?

5.Were confounding factors identified?

6. Were strategies established to deal with confounding factors?

7. Were the outcomes measured in a valid and reliable way?

8. Was an appropriate statistical analysis used?
